# Hidden in the urban parks of New York City: *Themira lohmanus*, a new species of Sepsidae described based on morphology, DNA sequences, mating behavior, and reproductive isolation (Sepsidae, Diptera)

**DOI:** 10.3897/zookeys.698.13411

**Published:** 2017-09-18

**Authors:** Yuchen Ang, Gowri Rajaratnam, Kathy FY Su, Rudolf Meier

**Affiliations:** 1 Lee Kong Chian Natural History Museum, Faculty of Science, National University of Singapore, 2 Conservatory Drive, Singapore 117377; 2 Evolutionary Biology Laboratory, Department of Biological Sciences, National University of Singapore, 14 Science Drive 4, Singapore 117543, Singapore

**Keywords:** cryptic species, Sepsidae, species description

## Abstract

New species from well-studied taxa such as Sepsidae (Diptera) are rarely described from localities that have been extensively explored and one may think that New York City belongs to this category. Yet, a new species of *Themira* (Diptera: Sepsidae) was recently discovered which is currently only known to reside in two of New York City’s largest urban parks. Finding a new species of *Themira* in these parks was all the more surprising because the genus was revised in 1998 and is not particularly species-rich (13 species). Its status is confirmed as a new species based on morphology, DNA sequences, and reproductive isolation tests with a closely related species, and is described as *Themira lohmanus* Ang, **sp. n.** The species breeds on waterfowl dung and it is hypothesized that this makes the species rare in natural environments. However, it thrives in urban parks where the public feeds ducks and geese. The mating behavior of *Themira lohmanus* was recorded and is similar to the behavior of its closest relative *T. biloba*.

## Introduction

Urban areas in industrialized countries are often considered species-poor and their biodiversity well-characterized for well-studied taxa. However, recent urban biodiversity scans have questioned this assumption and demonstrated that highly urbanized areas can contain significant numbers of hidden species. For example, the 2015 BioSCAN Malaise-trap study carried out in Los Angeles (California, USA) uncovered 40 new species in the mega-diverse Phoridae (Diptera) genus *Megaselia* Rondani, 1856 (Hartop et al. 2015, 2016). We recently discovered a new species of *Themira* Robineau-Desvoidy, 1830 (Diptera: Sepsidae) within New York City in Central Park and Prospect Park (New York, USA; hereafter, NYC). This discovery is surprising because unlike *Megaselia*, *Themira* is a small and well-studied genus in Sepsidae; its Nearctic fauna was recently revised by Ozerov (1998) and no new species were found. Our discovery is the first new *Themira* species described from the Nearctic in 90 years since *T. notmani* Curran, 1927.

The first sample of the new *Themira* species was collected in 2007 but originally identified as a new record of the Palearctic species *Themira biloba* Andersson, 1975 (Meier 2007). This identification was called in question when genetic data pointed to the presence of a cryptic species. Here we use an integrative taxonomy approach to confirm that the *Themira* material indeed belongs to a new species based on DNA sequences, morphology, and reproductive isolation tests with *T. biloba*. We then describe it as *Themira lohmanus* Ang, 2017. Similar to other new sepsid species that we have recently (re)described, we also cover life-history information such as mating behavior, breeding, and life-span (Ang et al. 2008; Ang et al. 2013; Tan et al. 2010) and discuss why a species in a well-studied genus such as *Themira* could have eluded discovery for such a long time.

## Materials and methods

### Material

Female *Themira* ‘*biloba*-like’ specimens were collected from Prospect Park, Brooklyn, NY, USA [40.6563 °N, 73.9686°W] in June 2015. *Themira* ‘*biloba*-like’ specimens are easily differentiated from other co-occurring *Themira* species [*T. flavicoxa* Melander *et* Spuler, 1917 and *T. minor* (Haliday, 1833)] based on their much larger size. Females from the new species were kept alive and fed with concentrated sugar water. Duck dung was also provided as breeding substrate. All dung was first frozen at -20°C for at least a week to prevent contamination from other species that may have already laid eggs in the dung. Additional specimens were used for morphological and DNA molecular analysis that were collected from Central Park (NYC, USA) in June 2006, along the shorelines of ‘Harlem Meer’ [40.7978°N, 73.9536°W]. These specimens were preserved in 70% EtOH.

### Morphological analysis

Ten specimens (five males and five females) from the culture established based on females obtained from Prospect Park as well as five male specimens from Central Park were selected for morphological analysis. They were first checked for intraspecific variation, and then compared to specimens from a *T. biloba* culture obtained from London (UK). One male and one female were imaged using the Dun Inc. Passport II Photomicrography imaging system (with 65mm MPE Canon Lens). Specimens were imaged extensively to capture as much morphology as possible so that character systems that may become important in the future have a higher chance of being serendipitously captured (Ang et al. 2013). Images were then processed in Photoshop CS5.

### Molecular analysis

COI barcode sequences of *ca.* 500 b.p. lengths were obtained for six specimens of the NYC population of the new species (One from Prospect Park and five from Harlem Meer), specimens representing three European populations of *T. biloba* and one specimen representing *T. putris* (Linnaeus, 1780) [Table 1, see Suppl. material 1 (.fasta format) for aligned barcodes]. COI was amplified using MTD4 and MTD9 using the PCR protocol as described in Su et al. (2008). These sequences were then aligned using MAFFT Ver. 7 (Katoh and Standley 2013). Uncorrected pairwise distances (see Srivathsan and Meier 2012) were used to quantify the intra- and inter-specific variability in SequenceMatrix (Vaidya et al. 2010), and SpeciesIdentifier (Meier et al. 2006) to cluster the sequences using thresholds of 1–5%.

**Table 1.** Details of specimens used in molecular analysis.

Specimen	Locality
*Themira putris*	Monterey, USA
*Themira biloba* “L”	London, UK
*Themira biloba* “Copen_III”	Copenhagen, DK
*Themira biloba* “Germany_K”	Munich, DE
*Themira* “*biloba*-like CP_I”	Central Park, NYC, USA
*Themira* “*biloba*-like CP_II”	Central Park, NYC, USA
*Themira* “*biloba*-like CP_III”	Central Park, NYC, USA
*Themira* “*biloba*-like CP_IV”	Central Park, NYC, USA
*Themira* “*biloba*-like CP_V”	Central Park, NYC, USA
*Themira* “*biloba*-like PP”	Prospect Park, NYC, USA

### Observations of mating behavior

In order to examine the mating behavior of the new species, virgin flies were obtained from the parental culture by rearing adults from a petri-dish with larvae-infested dung. Males and females were segregated within six hours of eclosion, and given five days to sexually mature (sepsid flies, at least in the *Themira* group acquire sexual maturity only after 2–5 days; Rajaratnam, pers. obs.). For the mating behavior observation, one virgin male was introduced into a small (3.5cm) petri-dish containing a single virgin female. Eleven mating trials were conducted. Behaviors were recorded at 5–15× magnification with a digital video recorder attached to a trinocular microscope (Leica Microsystema AG, Wetzlar, Germany). Recordings were started upon introduction of the virgin male and ended either upon a successful copulation, or after one hour if copulation did not occur. Recordings were then analysed frame-by-frame using the video editing software Final Cut Pro Ver.5 (Apple Inc., Cupertino, CA), and behavioral elements recorded.

### Determination of reproductive isolation

To examine the reproductive compatibility between the new species and a population of *T. biloba* from London, it was attempted to cross males from one with females from the other population. For this purpose, virgin males and females from both populations were obtained and combined into two mixed populations, each containing five males and five females from their respective populations (London ♂♂ x NYC♀♀ and vice versa; two replicates each). Control populations for each parental culture were used to confirm that the flies were fertile. Sugar water and dung was provided to all cultures and the dung checked daily for the presence of eggs and/or maggots.

## Results

### Morphological analysis

**Figure 1.**
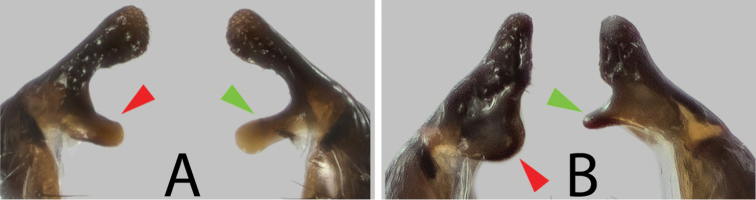
Surstyli (dorsal view) for male *Themira biloba* (**A**) and *Themira* “*biloba*-like” (**B**). Red arrows indicate basal process on left surstylus; green arrow for basal process on right surstylus.

The Prospect Park specimens were morphologically indistinguishable from the Central Park specimens, while specimens belonging to the new species were readily distinguishable from specimens belonging to the London population of *T. biloba* based on male genitalia: In male *T. biloba* specimens (Fig. 1A), males have symmetrical surstyli with a long, spatula-shaped process emerging basally on the surstylus (red and green arrows). In male *Themira* “*biloba*-like” specimens (Fig. 1B), the basal processes are asymmetrical: the left surstylus (red arrow) has a wide-based lamina, while the right surstylus (green arrow) has a pointed projection.

### Molecular analysis

Aligned COI sequences are shown in the Suppl. material 1 (.fasta format). Pairwise differences for these sequences calculated in SequenceMatrix (Vaidya et al. 2010) are detailed in Table 2: *Themira* “*biloba*-like” specimens have low intraspecific variation, with maximum observed COI distances of 0.2%, while the European *T. biloba* populations are more variable, with a largest difference of 0.9%. American *Themira* “*biloba*-like” differed with European *T. biloba* specimens by 4.46%, and with *T. putris* by 9.1%, which is higher than what is generally observed for intraspecific variation in Sepsidae (Zhao et al. 2013). Using SpeciesIdentifier (Meier et al. 2006), we found three clusters (representing *Themira* “*biloba*-like”, *T. biloba* and *T. putris*) at thresholds of 1 – 4% pairwise distance; *T. biloba* and *Themira* “*biloba*-like” clusters began to fuse at clustering threshold of 5% pairwise distance.

**Table 2.** COI barcode sequence pairwise differences for *Themira* “*biloba*-like”, *T. biloba*, and *T. putris* specimens, based on SequenceMatrix. Numbers shown are in percentage form.

	*T. putris* (Monterey, USA)	*T. biloba* (London, UK)	*T. biloba* (Copenhagen, DK)	*T. biloba* (Munich, DE)	*Themira* “*biloba*-like” PP (Prospect Park, NYC, US)	T. “*biloba*-like” CP_II (Central Park, NYC, US)	*Themira* “*biloba*-like” CP_III (Central Park, NYC, US)	*Themira* “*biloba*-like” CP_I (Central Park, NYC, US)	*Themira* “*biloba*-like” CP_IV (Central Park, NYC, US)	*Themira* “*biloba*-like” CP_V (Central Park, NYC, US)
*T. putris* (Monterey, USA)		9.1	9.1	9.1	9.1	9.1	9.1	9.1	9.1	9.1
*T. biloba* (London, UK)	9.1		0.9	0.9	4.46	4.46	4.46	4.46	4.46	4.46
*T. biloba* (Copenhagen, DK)	9.1	0.9		0.5	4.46	4.46	4.46	4.46	4.46	4.46
*T. biloba* (Munich, DE)	9.1	0.9	0.5		4.46	4.46	4.46	4.46	4.46	4.46
*Themira* “*biloba*-like” PP (Prospect Park, NYC, US)	9.1	4.46	4.46	4.46		0	0	0.2	0.2	0.2
T. “*biloba*-like” CP_II (Central Park, NYC, US)	9.1	4.46	4.46	4.46	0		0	0.2	0.2	0.2
*Themira* “*biloba*-like” CP_III (Central Park, NYC, US)	9.1	4.46	4.46	4.46	0	0		0.2	0.2	0.2
*Themira* “*biloba*-like” CP_I (Central Park, NYC, US)	9.1	4.46	4.46	4.46	0.2	0.2	0.2		0	0.2
*Themira* “*biloba*-like” CP_IV (Central Park, NYC, US)	9.1	4.46	4.46	4.46	0.2	0.2	0.2	0		0
*Themira* “*biloba*-like” CP_V (Central Park, NYC, US)	9.1	4.46	4.46	4.46	0.2	0.2	0.2	0.2	0	

### Reproductive isolation

No viable hybrid offspring were produced in the hybridization experiments although we observed intromission between the males and females in all experiments and controls almost immediately upon introducing the flies into the mating containers. Similarly, for all trials eggs were laid within two days in clutches of 10–20. Parental populations for both species produced larvae within three days, puparia within a week, and new adult flies within three weeks. However, the females from the hybridization laid eggs, but none of them hatched after ten days, and some had apparently started to rot because they were brown in color. No larvae, puparia or eclosed adults were observed even after three weeks and the trials were terminated after one month.

### Mating behavior observations

Eleven mating trials for the new species were conducted; only five were successful (≈ 45% mating success rate), and the average copulation time (= male intromittent) for these five trials was 1h 37m ± 18m (see Table 3 detailing the mating duration for the five successful trials). Nine discrete behavioral elements were observed for males and five for females (see Mating Behavior Profile, under the Species Description section). These 14 behavioral elements were compared with those of *Themira biloba*, for which five recorded mating trials for the Munich culture were available. No significant differences were found in mating behavior between the two species.

**Table 3.** Mating duration for the five (of eleven) successful mating trials. Note that pair 4 lacks intromittent and separation time as video recording was truncated during the mating experiment. Consequently, these two values, as well as those with a 0s are omitted from calculating average values.

	Pair 4	Pair 5	Pair 8	Pair 10	Pair 11	Average
**Time to mount**	24m 10s	0s (immediate mount)	5m 10s	0s (immediate mount)	9m 15s	**12m 48s (± 4m 28s)**
**Courtship time**	25m 42s	0s (immediate genital contact)	0s (immediate genital contact)	0s (immediate genital contact)	14m 32s	**20m 7s (±7m 16s)**
**Copulation time**	(truncated at 1h 16m)	1h 34m 42s	2h 2m 41s	1h 18m 21s	1h 34m 43s	**1h 37m 36s (±18m)**
**Separation time**	–	28s	20s	36s	21s	**26.5s (±6.41s)**

### Species concepts and taxonomic conclusions

Based on reproductive isolation experiments, it is shown that there is an endogenous, post-zygotic reproductive isolation mechanism that separates the *Themira* “*biloba*-like” from *T. biloba*, which renders it a discrete species from the latter based on the Biological (Mayer 2000) and Hennigian (Meier and Willmann 2000) species concepts. While there are no discernable differences in the mating behavior between these two species, *Themira* “*biloba*-like” has a unique set of morphological and mitochondrial molecular character differences that separate it from *T. biloba* and make it a discrete species as well under the Phylogenetic (*sensu* Wheeler and Platnick) species concept (Wheeler and Platnick 2000). In this way, we are employing an integrative taxonomic protocol *sensu* Schlick-Steiner et al. (2010) to test our hypothesis for a new species by using three lines of evidence from ‘independent disciplines’ (morphology, mitochondrial DNA and reproductive isolation data). While Schlick-Steiner et al. consider reproductive isolation and mitochondrial DNA data in the same category of “complementary” information, we argue that these two datasets are acquired through different disciplines, and are effectively independent of each other. The species is thus described as *Themira lohmanus* sp. n.

### Species description

#### Themira lohmanus

Taxon classificationAnimaliaORDOFAMILIA

Ang sp. n.

http://zoobank.org/19D6C4D4-9B1E-4649-9677-59A8254E3AA6

Figures 2 , 3

##### Material.

*Holotype.* ♂ [Lee Kong Chian Natural History Museum, Singapore (ZRC): ZRC_ENT_00001001], from ex-culture based on female collected June 2006 (Meier, R) in USA, New York, Brooklyn, Prospect Park [40.6563°N, 73.9686°W, elevation 20m ASL]. *Paratypes*. 2♂2♀ [ZRC: consecutive numbers running from ZRC_ENT_00001002 to ZRC_ENT_00001005], 3♂1♀ [American Museum of Natural History, New York, New York, USA (AMNH)], 1♂1♀ [National Museum of Natural History, Washington D.C., USA (USNM): USNMENT01384142, USNMENT01384143].

##### Etymology.

The new species is named after David J. Lohman, for his generous contributions of specimens to sepsid taxonomy.

##### Diagnosis.

*Themira lohmanus* is a relatively large, robust-looking sepsid species that resembles *T. biloba*. However, adult *T. lohmanus* males can be readily differentiated from the latter by their uniquely shaped, asymmetrical surstyli, which is symmetrical in *T. biloba* (Fig. 1A, see Morphological analysis section). While females of these two species do not have distinct structural differences, they can potentially be distinguished based on the color of the sclerous cuticle: in *T. biloba*, it tends to be glossy black while *T. lohmanus* tends to have a cupreous tinge. However, these characters may not be easily differentiated in faded specimens.

##### Description.

**Figure 2.**
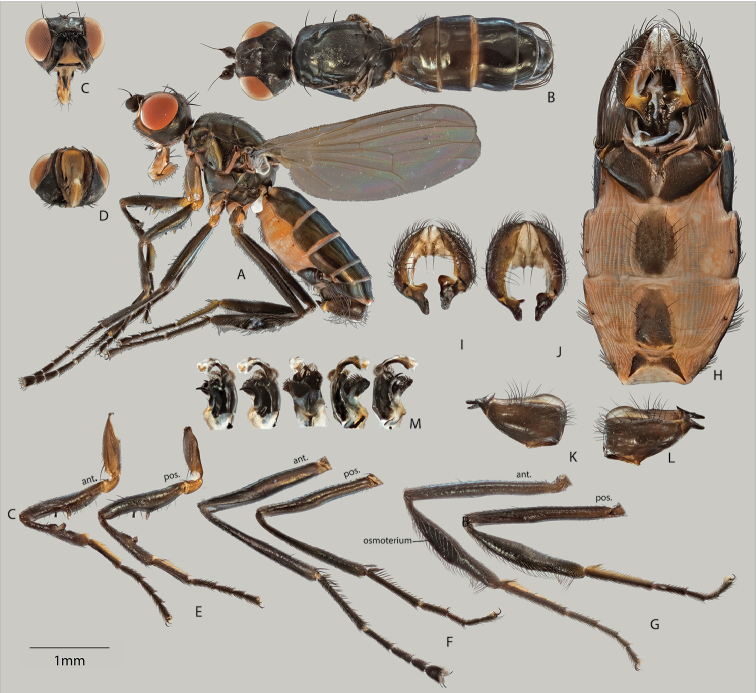
Adult male (**A–M**), showing lateral (**A**) and dorsal (**B**) views of habitus, anterior (**C**) and ventral (**D**) views of head capsule, anterior and posterior views of fore leg (**E**), mid leg (**F**) and rear leg (**G**); ventral view of abdomen (**H**) showing modified 4^th^ sternites; anterior (**I**), dorsal (**J**), left (**K**) and right (**L**) views of hypopygium, as well as various views of the penis (**M**).

**Figure 3.**
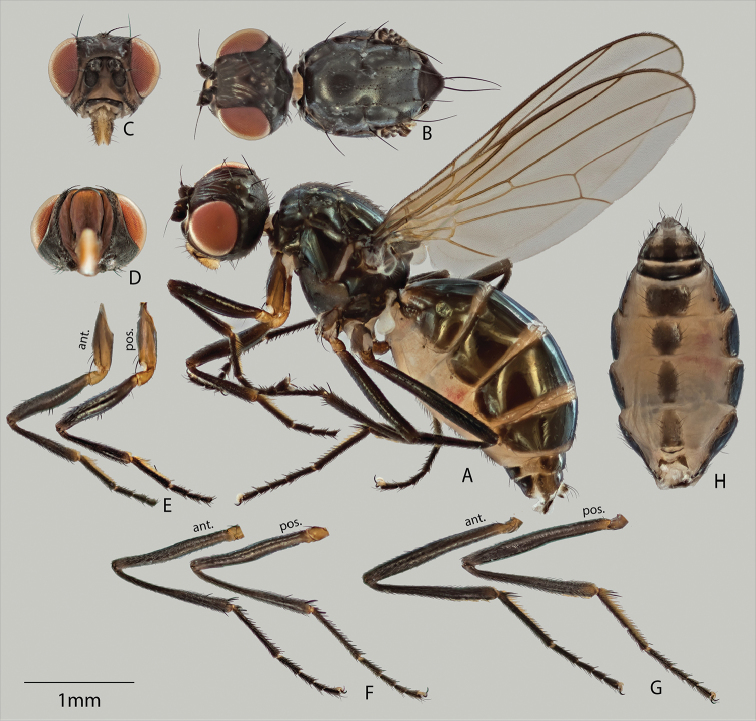
Adult female (**A–H**), showing lateral (**A**) and dorsal (**B**) views of habitus (*sans* abdomen), anterior (**C**) and ventral (**D**) views of head capsule, anterior and posterior views of fore leg (**E**), mid leg (**F**) and rear leg (**G**), and ventral view of abdomen (**H**).

**Males and females.** *Color* (Figures 2, 3). Adults are black-colored flies. Sclerites mostly black with a cupreous shiny tinge, while membranous cuticle with a variegated orange hue. Gena and face light brown. Trochanters, as well as posterior region of fore coxae yellow to light brown. Eyes red. Antennae all black with Wings clear, without pterostigma. Halteres and calypter white.

*Head* (A–D for Figures 2, 3). *Chaetotaxy.* Ocellar and postocellar setae divergent. Inner vertical setae convergent. 1 pair orbital setae, divergent. Posterior region of head capsule pruinose. Vibrissal angle with 2 larger vibrissae (dorsal longer than ventral), both smoothly medioclinate. Palps sclerotized and populated with multiple setae.

*Thorax* (A, B for Figures 2, 3). *Chaetotaxy*. Scutum with 1 pair discocentral setae on prescutum and 2 pairs on postscutum; anterior pair less than half as long as posterior pair. One postpronotal pair, 2 notopleural pairs; anterior pair half as long as posterior pair. One pair postalar setae. 1 pair apical scutellar setae. Anepisternum with short setulae on posterior region with 1 posteriad anepisternal seta. *Pruinosity pattern*. Scutum fully pruinose. Pleural thorax fully pruinose except for anepisternum, which is glossy.

*Wing* (Figures 2A, 3A). Veins bare. Cells entirely covered with microtrichiae except for basal costal cell. Section of costal vein between humeral vein and Radial 1 vein equally bisected by subcostal vein. Wing length 3.0–3.3 mm (♀), 3.2–3.8 (♂).

**Males.** *Legs* (Figure 2E–G). Figure 2E: Forefemur on ventral surface with 2–3 spines submedially, one large (yellowish, translucent) cuticular protrusion medially and one thick, blunt bristle postmedially. Foretibia on ventral surface with clasp-like cuticular protrusions medially, with 3-4 spines on anterior region of protrusions. Figure 2F: Mid-femur with 3 anterior spines medially, mid-tibia without distinct spines. Figure 2G: Rear-femur slightly curved, with 2-3 dorsal spines. Rear-tibia expanded medially to accommodate large, osmoteria that covers 2/3 of anterior side. All tarsi normal, with tarsomeres 1-4 proceedingly shorter.

*Abdomen* (Figures 2A, B, H). Figure 2B: Glossy tergites, with small short setulae until tergite 4; 5^th^ and 6^th^ tergites with stouter setae. Figure 2A: Abdominal spiracles with well sclerotized margins; spiracles 1-4 in membrane, 5 on margin of and 6 and 7 within tergite. Figure 2H: Sternite 4 as bi-lobed arms terminating in two tufts of long bristles each; desclerotized in the middle. Sternite 5 as a triangular keel that subducts under sternite 4 anteriorly.

*Hypopygium* (Figure 2I–L). Cerci a slight bump with one bristle each. Base of surstyli yellowish. Surstyli themselves asymmetrical; left surstylus as an enlarged lamina with a wide base, right surstylus as a shortened process.

**Females.** *Legs* (Figures 3E–G). Forefemur (Figure 3E) on ventral surface with 2-3 spines postmedially. Other legs unmodified. *Abdomen* (Figure 3A, H). Abdominal spiracles with well sclerotized margins; spiracles 1-4 in membrane, 5 on margin of, and 6 and 7 within tergite.

##### Distribution.

Nearctic. Thus far only found in New York City (Central Park and Prospect Park); likely to be found in more localities in the future, especially where waterfowl congregate.

##### Biology.

Similar to *T. biloba*, adults have only been found near water bodies, due to the association with waterfowl dung which they use for breeding. Under laboratory conditions they can breed in cow dung, but preferentially lay eggs in waterfowl dung. Eggs take 2–3 days to hatch, and feed as larvae for approximately 6–7 days before entering the pupal stage. Adult eclosion usually occurs after about another 7–8 days. Specimen longevity under laboratory conditions range from 1–3 months.

##### Mating behavior profile.

The mating behavior can be categorized into three sections: (1) approach and mount, (2) mounted courtship and copulation, and finally (3) separation. All described behaviors are shown in Video 1 (time given as mm:ss; YouTube link https://youtu.be/ZrtxN02zXLY). Our description is part of a larger series of papers describing and investigating the mating behavior of sepsids (e.g. Ang et al. 2008, 2013; Puniamoorthy et al. 2008, 2009; Tan et al. 2011, 2010). However, this is the first case in which a species that lacks species-specific behavioral elements.

##### (1) Approach and mount.

When a male detects and shows interest in a female, it immediately gives chase and will attempt to mount the female from the rear (Behavior A1: Male Approach and Mount – 00:02). This can happen almost immediately when the male is introduced into the female (e.g., pairs 5 and 10), or only after a period of time (e.g., pairs 4, 8, and 11).

##### (2) Mounted courtship and copulation.

The behavior varies between pairs. Some females (e.g., pairs 5, 8 and 10) may immediately accept genital contact with the male upon his mounting, and proceed directly to copulation. Other females may be more resistant to the male (e.g., pairs 4 and 11), and only accept genital contact after an average of ~ 20 minutes (± ~ 7m). Copulation time itself is ~ 1h 38m (± 18m). While mounted, the male will attempt to display nine types of courtship behaviors, as described in detail in Table 4. The first behavior is M1 (Male Foreleg-Female Wingbase Grasp – 00:11), but the grasp is released soon after. Behavior M2 (Male Dragging – 00:24) tends to occur in the earlier parts of courtship, especially when the male has just mounted the female. Behaviors M3 (Male Midleg Tarsal Curl – 00:32) and M4 (Male Wing Flutter – 00:43) are observed early in this section, but can also occur later prior to separation. The most prominent behavior set is M5-M8: The male will start with M5 (Male Midleg-Rearleg Rub – 00:51), which is likely used for transferring substances from its hind leg osmeterium to its mid legs (Araujo et al. 2014). The male will then directly proceed with either behavior M6 (Male Midleg-Female Wing Rub – 00:58), M7 (Male Midleg-Female Thorax Rub – 01:04) or M8 (Male Midleg-Female Head Rub – 01:10). Behavior M9 (Male Hind leg-Female Wing Rub – 01:16) was also observed in between the M5-8 behavior sets, and is also likely to be involved in the transfer of substances from hind leg osmeterium to wing (Araujo et al. 2014). These leg-rubbing behaviors are the most common actions performed by the male. Finally, the male will also perform M10 (Male Sternite Brushing – 01:25), which is always observed in the later part of this period, usually closer to the separation phase.

Females also display several behavioral elements, often in response to male behaviors. They are described in detail in Table 5: consistent is F1 (Female Body Shake – 01:51) when the male mounts her. This shaking may last for only a few seconds or is protracted; sometimes it is so violent that the couple will flip over. After her initial shake, the female tends to start walking around carrying the mounted male while the male may attempt to anchor his feet on the substrate (M2: Male Dragging; see above) resulting in the female dragging the male. The female is also observed to rub those parts of her body with her fore-, mid- and hind legs that the male has contacted (see behaviors M5 to M9) (F2: Female Self-rubbing – 02:04). Finally, the female may occasionally evert her ovipositor (F3: Female Ovipositor Eversion – 02:14). This only occurs during courtship, before the male is copulating with her.

##### (3) Separation.

Separation is always preceded by a significant amount of female shaking (Behavior F1). The male will then start to turn around (180°) facing directly away from the female. Both parties will start pulling (behavior S1 – 02:32) and after some amount of ‘straining’ (an average time of 26.5±6.41s), the pair will be able to disengage. This difficulty in separation is also known for *Themira biloba* and females have been observed to be dragging dead, intromittent males that have failed to disengage from the female (pers. obs. Mindy Tuan).

**Table 4.** Detailed descriptions of observed male behavioral elements during mating.

Behavior No.	Behavioral Element Name	Description of Behavioral Element
**M1**	Male Foreleg-Female Wing base grasp	Male uses ornamented forelegs to grasp on to female wing base. The male will not hold on for the duration of the mating, but release after a while (usually after the female is not shaking her body too much) and rest his forelegs on her thorax.
**M2**	Male Dragging	Male attempts to anchor on substrate with rear-legs, resulting in dragging by the female.
**M3**	Male Midleg Tarsal Curl	Male midleg is brought forward towards the female’s head, and the tarsi will curl laterally inwards towards her head. This action is repeated a few times before the midleg is brought backwards.
**M4**	Male Wing Flutter	Male flutters his wings while he brings them forward obliquely towards the female,
**M5**	Male Mid leg-Rear leg Rub	Male midleg first rubs against his own rear leg, before proceeding to either action M6, M7, or M9.
**M6**	Male Mid leg-Female Wing Rub	After performing action M5 (Male Midleg-rear leg rub), male will use midleg to rub on the female wing.
**M7**	Male Midleg-Female Thorax Rub	After performing action M5 (Male Midleg-rear leg rub), male will use midleg to rub against the female thorax and sometimes contacting the forelegs as well.
**M8**	Male Mid leg-Female Head Rub	After performing action M5 (Male Mid leg-rear leg rub), male will use midleg to rub against the female head capsule and sometimes contacting the antennae as well.
**M9**	Male Hind leg-Female Wing Rub	Male rear leg rubs the female wing-margin.
**M10**	Male Sternite Brushing	Male uses his sternite brush to rapidly tap the female abdomen ventrally.

**Table 5.** Detailed description of observed female behavioral elements during mating.

Behavior No.	Behavioral Element Name	Description of Behavioral Element
**F1**	Female Body Shake	Female shakes her body violently attempting to dislodge the mounted male.
**F2**	Female Self-rubbing	Female rubs own wings, head or forelegs, usually after a male has contacted that body part with his midleg.
**F3**	Female Ovipositor Eversion	Female everts her ovipositor.

## Discussion

New York City is one of the largest, most developed, and densely populated places on Earth (Florida et al. 2008), so that one may expect species discoveries here to be rare and unexpected: some of the more recent and prominent examples of species described from the city parks of NYC include the leopard frog *Rana kauffeldi* Feinberg & al., 2014, sweat bee *Lassioglossum gotham* Gibbs, 2011 and dwarf centipede *Nannarrup hoffmani* Foddai & al., 2002; these discoveries were all eventful to be featured in the New York Times (Foderado 2012; Olsen 2011; Stewart 2002). There is also public awareness and interest in city park biodiversity, as evidenced by the numerous “bioblitzes” in Central Park (Roach 2003; Woldan 2013). The discovery of *Themira lohmanus* sp. n. in Prospect Park is thus even more surprising, furthermore also because this species belongs to a well-studied genus of Sepsidae. In the Nearctic, Sepsidae only contain *ca.* 30 species across eight genera; *Themira* is the most species-rich genus with 13 species (Ozerov 1998). When *Themira* specimens were collected from Manhattan’s Central Park in 2016, they did not key to any of the 13 Nearctic species in Ozerov’s (1998) revision (one might note that Ozerov lists *T. mexicana* Ozerov, 1998 as a Nearctic species, but all material studied in his article were found within the Neotropical parts of Mexico). Using Pont and Meier (2002), these specimens were keyed to the distinctive *T. biloba*, which was previously unknown from the Nearctic region. However, subsequent DNA sequencing of the COI barcoding region revealed unexpectedly large genetic distances to *T. biloba* from Europe. In 2015 more specimens were collected from nearby Prospect Park, which prompted an integrative taxonomic investigation resulting in the current description of the new species.

*Themira lohmanus* exemplifies how little we know of our natural world even within densely populated cities. Urban areas are radically modified for human inhabitation and often degraded relative to natural conditions. Urban landscapes also tend to have different climates and host a variety of non-native and invasive species that often compete with the native biodiversity (McKinney 2002). While there is much evidence that urbanization is detrimental to invertebrate diversity and abundance (Davis 1976; 1978; Kotze and O’Hara 2003; McIntyre et al. 2001), not all taxa are adversely affected and certain synanthrophic taxa actually benefit (Dohmen et al. 1984; Elek and Lövei 2007; Magura and Tóthmérész 2004). This is because anthropogenic actions can produce an abundance of unique microhabitats that are rare under natural conditions. *Themira lohmanus* is likely to be one species that benefits from urbanization. Due to their association with water bodies and preference for waterfowl dung as a breeding substrate, urban ponds are likely to support the largest populations of this sepsid species. In natural environments, waterfowl have smaller population and defecation tends to be in the water thus making much of the feces unavailable for breeding by flies. However, in urban parks, waterfowl populations tend to be large and feeding on land which results in larger amounts of feces being dropped on moist soil where the dung provides optimal breeding conditions. This explains the large number of *Themira* individuals in European and North American city parks. However, changes in management practices can also quickly destroy desirable microhabitats. *Themira lohmanus* was not found in Central Park during a recent visit in 2015 although the species was present in 2006. This is likely because Central Park administration stopped the feeding of waterfowl and is now diligently cleaning the shoreline. Prospect Park is less ‘manicured’ and the ponds were found to still support populations of three *Themira* species. Overall, we believe that *T. lohmanus* used to be such a rare species that it was never collected in natural habitats. It only experienced a population boom after urban ponds were established and the population of New York became prosperous enough to start feeding waterfowl.

## Supplementary Material

XML Treatment for Themira lohmanus

## Supplementary materials

Supplementary material 1 Aligned COI sequences for *Themira putris*, *T. biloba*, and *Themira* '*biloba-like*' (i.e., *T. lohmanus* n. sp.) specimens Link: https://doi.org/10.3897/zookeys.698.13411.suppl1 Data type: Fasta file This dataset is made available under the Open Database License (http://opendatacommons.org/licenses/odbl/1.0/). The Open Database License (ODbL) is a license agreement intended to allow users to freely share, modify, and use this Dataset while maintaining this same freedom for others, provided that the original source and author(s) are credited.Yuchen Ang, Gowri Rajaratnam, Kathy FY Su, Rudolf Meier

## References

[B1] Ang Y Puniamoorthy N Meier R (2008) Secondarily reduced foreleg armature in *Perochaeta dikowi* sp.n. (Diptera : Cyclorrhapha : Sepsidae) due to a novel mounting technique. Systematic Entomology 33: 552–559. https://doi.org/10.1111/j.1365-3113.2008.00422.x

[B2] Ang Y Wong L Meier R (2013) Using seemingly unnecessary illustrations to improve the diagnostic usefulness of descriptions in taxonomy–a case study on *Perochaeta orientalis* (Diptera, Sepsidae). ZooKeys 355: 9–27. https://doi.org/10.3897/zookeys.355.6013 10.3897/zookeys.355.6013 PMC3867187 24363567

[B3] Araujo DP Tuan MJM Yew JY Meier R (2014) Analysing small insect glands with UV‐LDI MS: high‐resolution spatial analysis reveals the chemical composition and use of the osmeterium secretion in *Themira superba* (Sepsidae: Diptera). Journal of Evolutionary Biology 2: 144–1750. https://doi.org/10.1111/jeb.12420 10.1111/jeb.12420 24848999

[B4] Curran CH (1927) Four new American Diptera. American Museum Novitates 275: 1–4.

[B5] Davis BNK (1976) Wildlife, urbanisation and industry. Biological Conservation 10(4): 239–322. https://doi.org/10.1016/0006-3207(76)90002-1

[B6] Davis BNK (1978) Urbanisation and the diversity of insects. In: Mound LA Waloff N (Eds) Diversity of Insect Faunas. Blackwell, Oxford, 126–138.

[B7] Dohmen GP McNeill S Bell JNB (1984) Air pollution increases *Aphis fabae* pest potential. Nature 307: 52–53. https://doi.org/10.1038/307052a0

[B8] Elek Z Lövei GL (2007) Patterns in ground beetle (Coleoptera: Carabidae) assemblages along an urbanisation gradient in Denmark. Acta Oecologica 32: 104–111. https://doi.org/10.1016/j.actao.2007.03.008

[B9] Feinberg JA Newman CE Watkins-Colwell GJ Schlesinger MD Zarate B Curry BR Shaffer HB Burger J (2014) Cryptic Diversity in Metropolis: Confirmation of a New Leopard Frog Species (Anura: Ranidae) from New York City and Surrounding Atlantic Coast Regions. PLoS One 9. https://doi.org/10.1371/journal.pone.0108213 10.1371/journal.pone.0108213 PMC4212910 25354068

[B10] Florida R Gulden T Mellander C (2008) The rise of the mega-region. Cambridge Journal of Regions, Economy and Society 1: 459–476. https://doi.org/10.1093/cjres/rsn018

[B11] Foddai D Bonato L Pereira LA Minelli A (2003) Phylogeny and systematics of the Arrupinae (Chilopoda Geophilomorpha Mecistocephalidae) with the description of a new dwarfed species. Journal of Natural History 37: 1247–1267. https://doi.org/10.1080/00222930210121672

[B12] Foderado LW (2012) The New York Times: New Species in New York Was Croaking in Plain Sight. http://www.nytimes.com/2012/03/14/nyregion/new-leopard-frog-species-is-discovered-in-nyc.html [Accessed 2.I.2017]

[B13] Gibbs J (2011) Revision of the metallic Lasioglossum (Dialictus) of eastern North America (Hymenoptera: Halictidae: Halictini). Zootaxa 3073: 1–216.

[B14] Hartop EA Brown BV Disney HL (2015) Opportunity in our Ignorance: Urban Biodiversity Study Reveals 30 New Species and One New Nearctic Record for *Megaselia* (Diptera: Phoridae) in Los Angeles (California, USA). Zootaxa 3941(4): 451–484. https://doi.org/10.11646/zootaxa.3941.4.1 25947525 10.11646/zootaxa.3941.4.1

[B15] Hartop EA Brown BV Disney HL (2016) Flies from L.A., The Sequel: A further twelve new species of *Megaselia* (Diptera: Phoridae) from the BioSCAN Project in Los Angeles (California, USA). Biodiversity Data Journal 4. https://doi.org/10.3897/BDJ.4.e7756 10.3897/BDJ.4.e7756 PMC4867692 27226746

[B16] Katoh K Standley DM (2013) MAFFT Multiple Sequence Alignment Software Version 7: Improvements in Performance and Usability. Molecular Biology and Evolution 30: 772–780. https://doi.org/10.1093/molbev/mst010 23329690 10.1093/molbev/mst010 PMC3603318

[B17] Kotze DJ O’Hara RB (2003) Species decline – but why? Explanations of carabid beetle (Coleoptera, Carabidae) declines in Europe. Oecologia 135: 138–148. https://doi.org/10.1007/s00442-002-1174-3 12647113 10.1007/s00442-002-1174-3

[B18] Magura T Tóthmérész B (2004) Changes in carabid beetle assemblages along an urbanisation gradient in the city of Debrecen, Hungary. Landscape Ecology 19: 747–759. https://doi.org/10.1007/s10980-005-1128-4

[B19] Mayer E (2000) The Biological Species Concept. In: Wheeler QD Meier R (Eds) Species Concepts and Phylogenetic Theory: A Debate. Columbia University Press, New York, 17–29.

[B20] McIntyre NE Rango J Fagan WF Faeth SH (2001) Ground arthropod community structure in a heterogeneous urban environment. Landscape and Urban Planning 52: 257–274. https://doi.org/10.1016/S0169-2046(00)00122-5

[B21] McKinney DK (2002) Urbanization, biodiversity and conservation. Bioscience 52: 883–890. https://doi.org/10.1641/0006-3568(2002)052[0883:UBAC]2.0.CO;2

[B22] Meier R (2007) *Themira biloba* Andersson 1975 (Diptera: Sepsidae), a species from Manhattan’s Central Park that is new to the nearctic region. Journal of the New York Entomological Society 114: 176–177. https://doi.org/10.1664/0028-7199(2007)114[176:TBADSA]2.0.CO;2

[B23] Meier R Kwong S Vaidya G Ng P (2006) DNA Barcoding and Taxonomy in Diptera: a Tale of High Intraspecific Variability and Low Identification Success. Systematic Biology 55: 715–728. https://doi.org/10.1080/10635150600969864 17060194 10.1080/10635150600969864

[B24] Meier R Willmann R (2000) The Hennigian Species Concept. In: Wheeler QD Meier R (Eds) Species Concepts and Phylogenetic Theory: A Debate. Columbia University Press, New York, 30–43.

[B25] Olsen E (2011) The New York Times: City Bees Newly Discovered, Yet Here All Along. https://mobile.nytimes.com/images/100000001162714/blogs/cityroom/2011/11/10/bees/ [Accessed 2.I.2017]

[B26] Ozerov AL (1998) A review of the genus *Themira* Robineau-Desvoidy, 1830 (Diptera: Sepsidae) of the World, with a revision of the North American species. Russian Entomological Journal 7: 169–208.

[B27] Pont AC Meier R (2002) The Sepsidae (Diptera) of Europe. Brill, 221 pp.

[B28] Puniamoorthy N Ismail MRB Tan DSH Meier R (2009) From kissing to belly stridulation: comparative analysis reveals surprising diversity, rapid evolution, and much homoplasy in the mating behaviour of 27 species of sepsid flies (Diptera: Sepsidae). Journal of Evolutionary Biology 22: 2146–2156. https://doi.org/10.1111/j.1420-9101.2009.01826.x 19732260 10.1111/j.1420-9101.2009.01826.x

[B29] Puniamoorthy N Su K Meier R (2008) Bending for love: losses and gains of sexual dimorphisms are strictly correlated with changes in the mounting position of sepsid flies (Sepsidae: Diptera). BMC Evolutionary Biology 8: 155. https://doi.org/10.1186/1471-2148-8-155 10.1186/1471-2148-8-155 PMC2409323 18492287

[B30] Roach J (2003) National Geographic News: “BioBlitz” Finds 800-Plus Species in New York Park. http://news.nationalgeographic.com/news/2003/07/0708_030708_bioblitzresults_2.html [Accessed 2.I.2017]

[B31] Schlick-Steiner BC Steiner FM Seifert B Stauffer C Christian E Crozier RH (2010) Integrative Taxonomy: A Multisource Approach to Exploring Biodiversity. Annual Reviews of Entomology 55: 421–438. https://doi.org/10.1146/annurev-ento-112408-085432 10.1146/annurev-ento-112408-085432 19737081

[B32] Srivathsan A Meier R (2012) On the inappropriate use of Kimura-2-parameter (K2P) divergences in the DNA-barcoding literature. Cladistics 28: 190–194. https://doi.org/10.1111/j.1096-0031.2011.00370.x 10.1111/j.1096-0031.2011.00370.x 34861755

[B33] Stewart B (2002) The New York Times: A New Kind of New Yorker, One With 82 Legs. http://www.nytimes.com/2002/07/24/nyregion/a-new-kind-of-new-yorker-one-with-82-legs.html [Accessed 2.I.2017]

[B34] Su KF-Y Kutty SN Meier R (2008) Morphology versus molecules: the phylogenetic relationships of Sepsidae (Diptera: Cyclorrhapha) based on morphology and DNA sequence data from ten genes. Cladistics 24: 902–916. https://doi.org/10.1111/j.1096-0031.2008.00222.x 10.1111/j.1096-0031.2008.00222.x 34892884

[B35] Tan D Ng S Meier R (2011) New information on the evolution of mating behaviour in Sepsidae (Diptera) and the cost of male copulations *in Saltella sphondylii*. Organisms Diversity & Evolution 11: 253–261. https://doi.org/10.1007/s13127-011-0054-2

[B36] Tan DSH Ang Y Lim GS Ismail MRB Meier R (2010) From ‘cryptic species’ to integrative taxonomy: an iterative process involving DNA sequences, morphology, and behaviour leads to the resurrection of *Sepsis pyrrhosoma* (Sepsidae: Diptera). Zoologica Scripta 39: 51–61. https://doi.org/10.1111/j.1463-6409.2009.00408.x

[B37] Vaidya G Lohman DJ Meier R (2010) SequenceMatrix: concatenation software for the fast assembly of multi-gene datasets with character set and codon information. Cladistics 27: 171–180. https://doi.org/10.1111/j.1463-6409.2009.00408.x 10.1111/j.1096-0031.2010.00329.x 34875773

[B38] Wheeler QD Platnick NI (2000) The Phylogenetic Species Concept (sensu Wheeler and Platnick). In: Wheeler QD Meier R (Eds) Species Concepts and Phylogenetic Theory: A Debate. Columbia University Press, New York, 55–69.

[B39] Woldan O (2013) Scientific American Blog: Biology in the Big Apple: Surveying the Wildlife of Central Park. https://blogs.scientificamerican.com/guest-blog/biology-in-the-big-apple-surveying-the-wildlife-of-central-park/ [Accessed 2.I.2017]

[B40] Zhao L Ang A Amrita S Su KF-Y Meier R (2013) Does better taxon sampling help? A new phylogenetic hypothesis for Sepsidae (Diptera: Cyclorrhapha) based on 50 new taxa and the same old mitochondrial and nuclear markers. Does better taxon sampling help? A new phylogenetic hypothesis for Sepsidae 69: 153–164. https://doi.org/10.1016/j.ympev.2013.05.011 10.1016/j.ympev.2013.05.011 23707858

